# Education and advocacy in acute kidney injury in children: a call for action

**DOI:** 10.1590/2175-8239-JBN-2023-E016en

**Published:** 2023-12-01

**Authors:** Marcelo de Sousa Tavares

**Affiliations:** 1 Santa Casa de Belo Horizonte, Belo Horizonte, MG, Brazil.

Acute kidney injury (AKI) has been a major issue in pediatric nephrology over the last two decades. The increase in cases related to a variety of conditions, such as human stem cell transplantation, trauma, cardiac surgery, and sepsis, among others, has led to the development of technology that offers better treatment options for these patients.

A better understanding of the pathophysiological mechanisms underlying AKI and the early diagnosis and staging of this condition in the intensive care unit (ICU) have led to the development of biomarkers. Additionally, the role of fluid balance in clinical outcomes has been established.

The presence of pediatric nephrologists in ICUs and kidney replacement therapies are considered essential in intensive care medicine. In the late 1990s, the number of pediatric ICUs increased proportionally more than the pediatric population, as described by Randolph et al.^1^. However, pediatric nephrologists were never available in 33.5% of the ICUs participating in the survey. This observation was probably associated with a shortage of pediatric nephrologists in comparison to other specialties, e.g., pediatric intensivists, as pointed out by Ashoor et al.^2^.

The importance of pediatric nephrologists extends beyond clinical care and plays an important role in pediatric medical education, not only for medical students but also for pediatric intensive care residents, nephrologist, and the entire healthcare team^3^.

Education regarding AKI must be part of the curriculum and training for all healthcare professionals, as stated recently by Goldstein et al.^4^ in a Consensus of the 26th Acute Disease Quality Initiative Meeting, the 1^st^ pediatric ADQI. The key components of AKI education include training of healthcare workers at all levels, context-appropriate educational programs, and the development of appropriate checklists and clinical practice guidelines.

Differences about AKI management and expected outcomes vary among specialists. In a nation-wide study by Che et al.^5^, web-based surveys were sent to Canadian pediatric nephrologists and pediatric intensivists via professional listservs. Interestingly, the prescription of renal replacement therapies varied by professional group: hemodialysis was mostly prescribed by nephrologists (65%), and a mix of nephrology, ICU, or a shared nephrology-ICU model for peritoneal dialysis and continuous renal replacement therapy (CRRT) (59%) by intensivists. Nephrologists were more likely than pediatric ICU physicians and nurses to recommend long-term follow-up for patients who develop any AKI during ICU stay.

Ferrari et al.^6^ retrospectively analyzed how the interaction between pediatric nephrologists and intensivists influenced the frequency of dialysis indication, fluid balance grading, and pRIFLE over 2 sequential observation periods (before and after 2013) in a tertiary pediatric intensive care unit in Brazil. The main intervention was discussion of cases by the pediatric nephrologist on a regular basis and discussion of common topics regarding AKI and nephrointensivism. The authors concluded that a significant decrease in the number of indications for dialysis/year, decrease in infusion volume, increase in dialysis duration, and improvement in the discrimination of the pRIFLE diuresis component in AKI development were observed. However, an important hard outcome parameter, death rate, did not change over the 2 analyzed time intervals. The retrospective analysis based on medical records is a limitation of the study.

A major merit of the work by Ferrari et al.^6^ is to raise awareness of the paucity of studies about the impact of pediatric nephrologists and interaction with intensivists. Goldstein et al.^4^ highlighted a lack of research on the effects of education initiatives aimed at the public, patients, and professionals on AKI. Further studies on the role and impact of pediatric nephrologists in pediatric ICUs are warranted.
